# Nuclear Trafficking of Retroviral RNAs and Gag Proteins during Late Steps of Replication

**DOI:** 10.3390/v5112767

**Published:** 2013-11-18

**Authors:** Matthew S. Stake, Darrin V. Bann, Rebecca J. Kaddis, Leslie J. Parent

**Affiliations:** 1 Division of Infectious Diseases and Epidemiology, Department of Medicine, Penn State College of Medicine, 500 University Drive, Hershey, PA 17033, USA; E-Mails: mss31@psu.edu (M.S.S.); dbann@hmc.psu.edu (D.V.B.); rjk297@psu.edu (R.J.K.); lparent@psu.edu (L.J.P); 2 Department of Microbiology & Immunology, Penn State College of Medicine, 500 University Drive, Hershey, PA 17033, USA

**Keywords:** nuclear entry, nuclear export, nucleocytoplasmic trafficking, virus assembly, viral RNA packaging, retroviral Gag protein, HIV-1 Rev protein, Rous sarcoma virus, retroviruses

## Abstract

Retroviruses exploit nuclear trafficking machinery at several distinct stages in their replication cycles. In this review, we will focus primarily on nucleocytoplasmic trafficking events that occur after the completion of reverse transcription and proviral integration. First, we will discuss nuclear export of unspliced viral RNA transcripts, which serves two essential roles: as the mRNA template for the translation of viral structural proteins and as the genome for encapsidation into virions. These full-length viral RNAs must overcome the cell’s quality control measures to leave the nucleus by co-opting host factors or encoding viral proteins to mediate nuclear export of unspliced viral RNAs. Next, we will summarize the most recent findings on the mechanisms of Gag nuclear trafficking and discuss potential roles for nuclear localization of Gag proteins in retrovirus replication.

## 1. Introduction: Nuclear Trafficking Events in Retrovirus Replication

**Figure 1:**
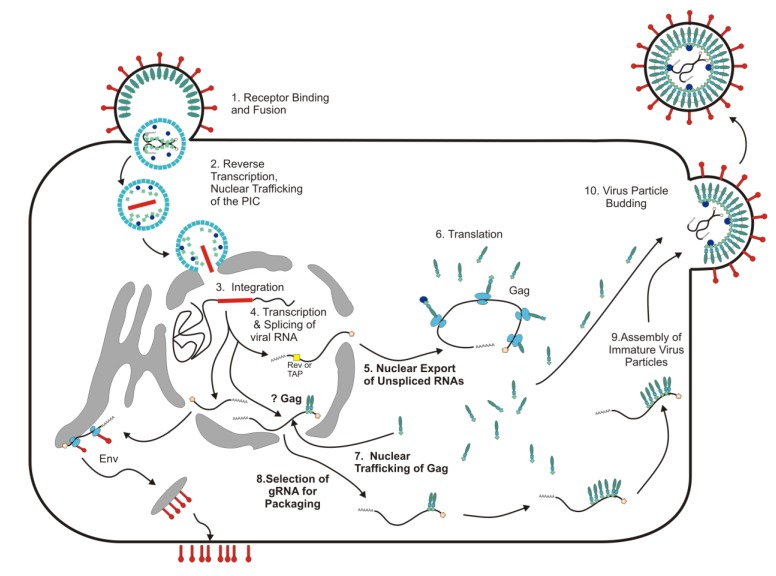
Model of retrovirus replication. Retroviral infection is initiated with binding of the viral Env protein to a cell surface receptor and fusion of the viral envelope with the cellular membrane (step 1). The viral RNA genome is reverse transcribed from RNA to DNA (step 2) to form the provirus, which is stably integrated into the genome of the host cell (step 3). Viral RNA is transcribed by the host polymerase II, and a portion of the RNA is spliced to direct the translation of the Env glycoprotein and other viral proteins (step 4). A portion of the viral RNA remains unspliced and is exported from the nucleus by the host factor TAP/NXF1 (e.g., simple retroviruses MPMV and RSV) or virally encoded Rev-like proteins (e.g., complex retroviruses like HIV-1 and HTLV-I) (step 5) to serve as a template for Gag and Gag-Pol translation (step 6). The Gag proteins of some retroviruses traffic through the nucleus during assembly (step 7). It has been postulated that the nuclear population of Gag (denoted by “?”) might select genomic RNA (gRNA) and transport it into the cytoplasm for packaging. Alternatively, selection of genomic RNA may occur in the cytoplasm (step 8). In either case, the Gag-gRNA complex is transported to the plasma membrane (step 9) where additional Gag molecules bind the viral RNP to complete assembly of the immature virus particle, which buds from the plasma membrane (step 10). The steps in replication that are covered in this review are indicated in bold. Figure modified from [22] and used with the author’s permission.

Retroviruses interact with nuclear trafficking machinery during several different phases of their replication cycles (Figure 1). Retrovirus replication has been divided into “early” and “late” stages, with early events extending from virus entry through integration and late stages encompassing expression of viral RNA from the provirus through virus assembly and budding of immature virus particles from the host cell. During early infection, all retroviruses must gain access to the host chromatin for the provirus to integrate. Most retroviruses depend on mitosis and breakdown of the nuclear envelope to undergo integration. However, lentiviruses like human immunodeficiency virus type 1 (HIV-1) infect non-dividing cells, having developed strategies to enter the nucleus by passing through intact nuclear pores (reviewed in [1,2,3]). Although several viral factors, including HIV-1 MA (matrix), IN (integrase), Vpr, and the reverse-transcribed proviral DNA have been implicated in nuclear entry of the pre-integration complex (PIC), more recent data indicate that the CA (capsid) protein, nuclear import factor transportin-3 (TNPO3), and the nucleoporin Nup358 are important determinants [4,5,6,7,8,9,10,11,12,13,14,15]. Thus, retroviruses have evolved different strategies to promote proviral integration through complex interactions between host and viral factors. Although these early events are essential to establish retroviral infection, this review will focus primarily on interactions of retroviruses with nuclear transport machinery following integration.

Once integration is completed, viral RNA is synthesized by the cellular RNA polymerase II. Retroviral transcripts are co-transcriptionally modified with the addition of a 5’ cap and 3’ poly(A) tail, like cellular mRNAs. A fraction of the viral RNA is spliced and exported out of the nucleus to serve as mRNA for translation into viral proteins. The remainder of the viral RNA remains unspliced and must be transported from the nucleus into the cytoplasm where it serves two functions: (i) as the template for translation of the viral structural proteins, Gag and Gag-Pol; and (ii) as the viral genome, which is packaged into virus particles. Because export of unspliced and incompletely spliced cellular RNAs is prevented by host machinery to prevent translation of abnormal proteins (reviewed in [17]), retroviruses must circumvent this cellular blockade to export their full-length RNA molecules (reviewed in [16,18]). Prior translation of the unspliced viral RNA is not a prerequisite for genome encapsidation, as genomic RNA can be packaged in *trans* (reviewed in [16]). The genomic RNA forms a noncovalent dimer and is encapsidated through an interaction between the psi (ψ) packaging sequence near the 5’ end of the genome and the NC (nucleocapsid) domain of the Gag protein. 

The Gag protein, which directs the assembly and budding of virus particles from the plasma membrane, is localized primarily in the cytoplasm of infected cells. However, the Gag proteins of Rous sarcoma virus (RSV), feline immunodeficiency virus (FIV), mouse mammary tumor virus (MMTV), prototype foamy virus (PFV), murine leukemia virus (MLV), Mason-Pfizer monkey virus (MPMV), HIV-1, and several retrotransposons undergo nuclear localization under certain conditions (Table 1). The RSV, FIV, and PFV Gag proteins utilize the cellular CRM1 protein for nuclear export [19,20,21], but the host importins involved in nuclear import of Gag have only been defined for RSV. In this review, we will focus on nuclear transport events associated with the nucleocytoplasmic trafficking of unspliced retroviral RNAs and Gag proteins and their roles in virion assembly. 

**Table 1:** Retroviral proteins that undergo nuclear trafficking*

Retroviral Protein	Localization of the Population Associated with the Nuclear Compartment	Nuclear Localization Mechanism	Nuclear Export Mechanism
*Alpharetrovirus*			
RSV Gag	Nucleus [ 20,23] Punctate Nuclear Foci [23,24] Nucleoli [24]	Imp11 (MA domain) [ 25,26] TNPO3 (MA Domain) [26] Importin α/β (NC Domain) [25,26]	CRM1 [ 20,25]
RSV NC	Nucleoli [24]	Importin α/β [25,26]	None
RSV RT, β subunit	Nucleus [27]	Unknown	None
RSV IN	Nucleus [28]	Shares Import Pathway with Histone H1 [29]	None
*Betaretrovirus*			
MMTV Gag	Nucleoli [30]	Unknown [30]; Nucleolar Localization Increased with RPL9 Overexpression [30]	No Identified NES
MMTV NC	Nucleoli [24,30]	Unknown;	None
MMTV Rem	Nucleoli [31]	Retrotranslocation from Endoplasmic Reticulum to Nucleus [32]	CRM1 [31]
MPMV Gag	Nuclear Pore Complex; Low Levels in Nucleus [33,34]	Unknown; Nuclear localization Increased with Ubc9 Overexpression [34]	CRM1 [35]
JSRV Rej	Nucleus and Nucleoli [36,37]	Unknown	Rej Function Depends on CRM1 [37]
*Gammaretrovirus*			
MLV Gag	Nucleus [38]	Unknown	No Identified NES
MLV NC	Nucleus [24,39] Nucleoli [24,39]	Unknown	None
MLV p12	Mitotic Chromatin [40,41]	Unknown	None
MLV IN	Nucleus and Nucleoli [39]	Unknown; Interacts withBrd4 in Nucleus [42]	None
*Deltaretrovirus*			
HTLV Rex	Nucleus and Nucleoli [43,44,45] Punctate Nuclear and Nucleolar Foci [43]	Importin β [46]	CRM1 [47,48]
BLV Rex	Punctate Nuclear Foci [49] Nuclear Pore Complex [49]	Unknown	CRM1 [49]
*Lentivirus*			
HIV-1 Gag	Nucleolar [24]	Unknown	Not CRM1 [19,35,50]
HIV-1 IN	Nucleus [14,51,52,53,54,55]	Importin α3 [51] Importin α [14,52] Importin 7 [56]	None
HIV-1 NC	Nucleoli [24] Nucleus [57,58]	Unknown	None
HIV-1 Rev	Nucleus andNnucleoli [44,59] Nuclear Foci [59] HIV-1 Transcription Sites [60]	Importin β [61,62] Transportin, Importin 5, and Importin 7 [63]	CRM1 [47,64]
HIV-1 Vpr	Nucleus [65,66] Nuclear Pore Complex [6,66]	Importin α [66] Interacts Directly with Nuclear Pore Complex [67,68]	CRM1 [65]
HIV-2 Rev	Nucleoli [69]	Unknown	Unknown
HIV-2 Vpx	Nucleoplasm [70,71]	Unknown	No Identified NES
FIV Gag	Nucleus [19], Nucleoli [19], and Nuclear Envelope [72]	Unknown	CRM1 [19]
FIV Rev	Nucleolus [73]	Unknown	CRM1 [74]
EAIV Rev	Nucleus [74]	Unknown	CRM1 [74]
BIV Rev	Nucleus and Nucleoli [75,76]	Importin α [77]	CRM1 [77]
MVV Rev	Nucleoli [78]	Unknown	Unknown
CAEV Rev	Nucleoli [79,80]	Unknown	Unknown
*Spumaretrovirus*			
PFV Gag	Nucleus [21,81] Punctate Nuclear Foci [82,83] Mitotic Chromatin [82]	Binds to H2A/H2B on Mitotic Chromatin [82,83]	CRM1 [21]

* Note: Transcriptional activators related to HIV-1 Tat were not included in the table.

## 2. Nuclear Export of Unspliced and Incompletely Spliced RNAs of Complex Retroviruses

Productive retroviral infection requires unspliced viral transcripts to be transported into the cytoplasm where they are translated into the essential viral proteins Gag and Gag-Pol. To circumvent intrinsic cellular blockades that prevent the export of incompletely spliced RNAs from the nucleus, complex retroviruses encode *trans*-acting viral proteins that export their intron-containing viral RNAs from the nucleus. HIV-1 Rev was the first member of this family to be discovered; however, Rev-like proteins have been described in the *Lentivirus* [e.g., Rev proteins of human immunodeficiency virus type-2 (HIV-2), simian immunodeficiency virus (SIV), FIV, equine infectious anemia virus (EIAV), bovine immunodeficiency virus (BIV), Maedi-visna virus (MVV) and caprine encephalitis-anemia virus, CAEV)] [73,84,85,86,87,88,89,90,91,92,93], *Deltaretrovirus* [(e.g., Rex proteins of human T cell leukemia virus type-I (HTLV-I) and bovine leukemia virus (BLV)], and *Betaretrovirus* [e.g., Rem protein of MMTV and Rej protein of Jaagsiekte sheep retrovirus (JSRV)] genera [31,36,37,77,94,95,96]. Rev-like proteins localize to the nucleus and nucleolus through interactions with a variety of import factors (see (1), and they contain CRM1-dependent nuclear export signals (NESs) [45,47,77,97,98,99,100]. 

HIV-1 Rev recognizes and binds to the highly structured *cis*-acting Rev-responsive element (RRE) in HIV-1 RNAs [101,102,103,104] and undergoes multimerization, which is important for its export function (reviewed in [105]). Multimerization of Rev was demonstrated within the nucleolus in living cells, suggesting that the nucleolus may be the site of Rev multimer formation [106]. However, the relevance of these experiments is somewhat limited because they were not performed in the context of HIV-1 infection, nor was the RRE sequence expressed in the cells. Attempts to define whether nucleolar localization of Rev is important for binding to RRE-containing RNAs have been difficult because the Rev RNA binding domain overlaps with the nuclear/nucleolar localization signal. However, HIV-1 unspliced RNA appears to undergo nucleolar trafficking based on the finding that the RNA is cleaved by ribozymes artificially targeted to the nucleolus [107] and small nucleolar RNAs engineered to contain the RRE are exported into the cytoplasm by Rev [108,109]. Together, these data provide evidence that Rev and the RRE-containing viral transcripts both traffic through the nucleolus, but there is no definitive evidence that nucleolar trafficking of the Rev-RRE complex is essential for nuclear export of HIV-1 RNA during natural virus infection. 

In the nucleoplasm, Rev co-localizes with the SR-domain splicing factor SC-35 in nuclear speckles [59,110], intrachromatin granule clusters enriched in mRNA splicing factors and snRNPs located adjacent to sites of active transcription [111,112,113,114,115]. The association of Rev with splicing factors in speckles suggests that Rev binds to the RRE co-transcriptionally, just as splicing factors bind to cellular transcripts as they are synthesized [116]. In support of this idea, previous studies demonstrated that the Rev-RRE interaction is abrogated by transcription inhibitors [117]. More recently, it was shown that Rev co-localizes with HIV-1 RNA at transcription sites, providing strong evidence that Rev binds RRE-containing transcripts co-transcriptionally [60]. 

HIV-1 Rev mediates export of unspliced viral RNAs through an interaction between the NES of Rev and the CRM1/RanGTP export complex. However, many other cellular proteins also interact with Rev, indicating that nuclear export of unspliced HIV-1 RNA depends upon a complex network of interactions [60,62,105,118,119,120,121,122,123,124,125,126,127]. For example, several RNA helicases interact with Rev, including DDX1 and DDX3, which help to maintain Rev localization in the nucleolus and nucleus [124,126,127,128]. In addition, the nuclear matrix-associated protein Matrin 3 binds HIV-1 RNA co-transcriptionally and facilitates Rev-mediated nuclear export of unspliced RNA [60,129]. Although the precise mechanism by which Matrin 3 facilitates HIV-1 RNA export has not been elucidated, Matrin 3 increases the stability and expression of cellular mRNAs, suggesting that it may have a similar effect on HIV-1 RNA [130]. The finding that Matrin 3 interacts with HIV-1 RNA also creates an intriguing connection between HIV-1 RNA export and the nuclear matrix, which not only provides structural support to the nucleus [131] but also associates with areas of euchromatin involved in ongoing transcription [132]. Recent evidence also suggests that components of the nuclear matrix called nuclear regulatory networks bind genomic DNA and form a tubular pathway leading to nuclear pore complexes for nuclear export of transcripts and proteins [133,134]. Therefore, it is intriguing to postulate that Matrin 3 bridges the interaction between Rev and active HIV-1 RNA transcription sites [60,129,135,136], recruiting the CRM1 nuclear export machinery associated with nuclear regulatory networks to transport viral ribonucleoprotein complexes (RNPs) through the nuclear pore and into the cytoplasm.

## 3. The Role of *cis*-Acting Sequences in RNA Export of Simple Retroviruses

In contrast to complex retroviruses that encode *trans*-acting factors to facilitate nuclear export of unspliced RNA, simple retroviruses have evolved *cis-*elements to circumvent the blockade to export of unspliced transcripts from the nucleus. MPMV and other type D retroviruses, including simian retrovirus-1 and 2 (SRV-1 and SRV-2), contain a small *cis* element, the constitutive transport element (CTE), which is required for nuclear export of unspliced viral RNA [137,138]. When inserted into unspliced or incompletely spliced HIV-1 transcripts, the MPMV CTE sequence replaces the function of the Rev/RRE complex, leading to expression of Gag and Env followed by the production of infectious virus particles [137]. Thus, Rev/RRE and the CTE provide similar roles in the nuclear export of unspliced RNA in complex and simple retroviruses.

Insight into the mechanism by which CTE-containing RNAs are exported from the nucleus was provided by proteomic studies that identified the host nuclear export protein Tip-associating protein/Nuclear RNA export factor 1 (TAP/NXF1) as a binding partner of CTE complexes [139,140]. Microinjection of *Xenopus* oocyte nuclei expressing TAP/NXF1 and an intron containing the CTE resulted in nuclear export of the RNA in the absence of splicing [141,142]. The TAP/NXF1 protein, homologous to the mRNA export protein Mex67p in yeast, forms a heterodimer with NXT1 to transport mRNAs out of the nucleus [139,140,143,144,145]. The N-terminal domain of TAP/NXF1 contains an RNA recognition motif that binds to a structured stem-loop in the CTE, inducing structural changes in both TAP/NXF1 and the CTE-containing RNA to promote nuclear export of the viral RNP [146]. Mutations in the RNA or in the coding region of TAP/NXF1 that disrupt CTE-TAP/NXF1 complex formation prevent expression of CTE-containing reporters *in vivo* [146]*.*

A putative *cis*-acting unspliced RNA transport element was also identified in RSV, which lies within 115 nucleotide direct repeat (DR) sequences flanking the *v-src* oncogene [147]. DR elements are highly conserved in avian retroviruses [148], and strains missing the *src* sequence maintain at least a single DR element to remain replication-competent [149,150]. The biological role of the DR elements is complex; pleotropic, contradictory effects on virus replication have been reported, including differences in levels of cytoplasmic accumulation of RSV RNA, viral RNA stability, expression of the Gag polyprotein, viral RNA packaging and virus assembly [148,151,152,153]. These conflicting results may be explained by differences in cell types or the use of subviral reporter constructs in some studies and full-length, replication-competent viruses in others. 

RSV RNAs containing the DR elements are exported by the cellular mRNA export factor TAP/NXF1 and the RNA helicase Dbp5 [139,154,155]. An additional host factor may bridge the interaction because neither TAP/NXF1 nor Dbp5 bind the DR element directly. Because the RSV Gag protein was reported to traffic through the nucleus [20], LeBlanc *et al*. used a subviral reporter construct containing either the DR sequence or the ψ sequence to examine whether Gag could enhance translation by promoting nuclear export of unspliced RNA [155]. Gag did not enhance translation of the reporter; however, nucleocytoplasmic fractionation of the RNA was not performed, so it is unclear whether Gag had an effect on cytoplasmic levels of the DR- or ψ-containing RNAs. Thus, these experiments suggest that DR elements mediate nuclear export through TAP/NXF1 and Dbp5 to stimulate translation of RSV unspliced RNA, but Gag is not likely to be involved in DR-mediated RNA transport. 

Taking the available data into account, we postulate that there is a temporal switch in RSV replication, such that viral transcripts produced early after integration are exported using DR-mediated interactions with Tap/NXF1 and Dbp5 to initiate the synthesis of Gag and GagPol proteins. We hypothesize that as the levels of these viral structural proteins increase, the Gag protein enters the nucleus where it may bind unspliced viral RNA and export it into the cytoplasm for encapsidation into virions [20,156]. It is possible that other simple retroviruses may use a similar mechanism. We speculate that MPMV RNA export could be similarly regulated, since a subpopulation of MPMV Gag localizes to the nucleus and nuclear envelope, and the pp24 domain NLS has been linked to genomic RNA incorporation [33,34,35]. Complex retroviruses regulate this temporal switch between early and late gene expression differently; the Rev protein is synthesized from a fully spliced mRNA and then traffics into the nucleus to promote nucleocytoplasmic transport of unspliced RNAs for structural gene expression and genome packaging. 

## 4. Foamy Viruses Use a Unique Pathway among Retroviruses for Nuclear Export of Viral RNAs

FVs, members of the genus *Spumavirus*, share similarities with both simple and complex retroviruses, yet they have several unique characteristics (reviewed in [157,158]). Unlike other retroviruses, Gag is the only protein translated from an unspliced transcript [159,160,161]. Additionally, instead of being expressed as a fusion protein with Gag using frameshifting or termination codon suppression, Pol is expressed from a separate spliced viral mRNA [162]. Similar to complex retroviruses however, FVs encode the transcriptional transactivator protein Tas, which functions analogously to HIV-1 Tat [163]. FVs do not encode an accessory protein with Rev-like functionality [164], therefore, FVs rely entirely on host factors to mediate the export of unspliced RNA from the nucleus, similar to the simple retroviruses MPMV and RSV. 

Among retroviruses, PFV utilizes a unique set of cellular factors that bind to its viral RNA for nucleocytoplasmic transport. Rather than using TAP/NXF1, like the *cis-*acting RSV and MPMV RNA transport elements [139,155], spliced and unspliced PFV RNAs interact with host proteins HuR and ANP32A/B to exit the nucleus through the CRM1 pathway [165]. HuR associates with PFV RNAs and the adaptor proteins ANP32A or ANP32B, which bridge the association with CRM1 [165,166,167]. The HuR-interacting RNA sequence likely resides in the 3’ region of the genome, which is shared among spliced and unspliced PFV RNAs [165], although the PFV 3’ end does not share homology with previously-characterized RNA sequences that bind HuR [165]. Although both the spliced and unspliced PFV transcripts appear to be exported by the same pathway, subsequent interaction with the cytoplasmic mRNA processing factor DDX6 may distinguish the genomic RNA from the viral transcripts earmarked for translation on polysomes [168]. It is not yet known whether all PFV RNPs exported by HuR-ANP32A/B-CRM1 traffic to the same subcellular location where DDX6 co-localizes with genomic RNA in association with Gag. However, having spliced and unspliced RNAs targeted to the same cytoplasmic localization may not pose a problem for selective genomic RNA selection by PFV Gag because both segments of the bipartite *cis* packaging signal are present only on unspliced viral RNA [169].

## 5. Nuclear Trafficking of Retroviral Gag Proteins: RSV as the Prototype

**Figure 2:**
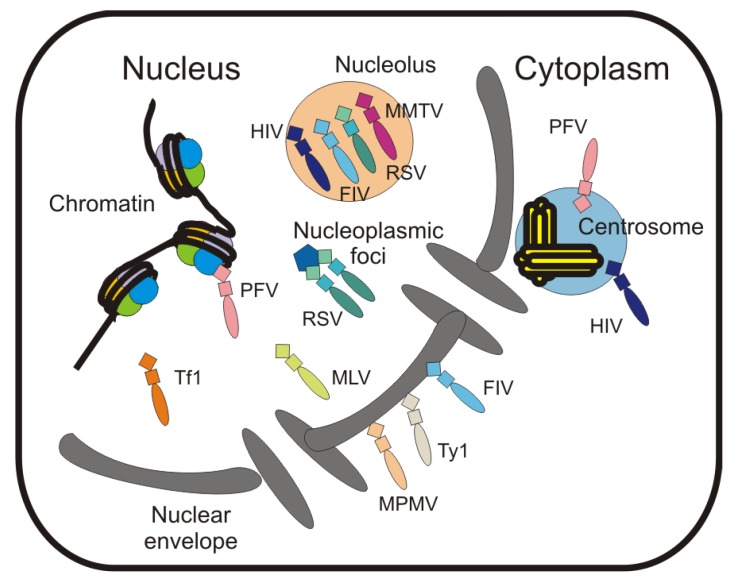
Localization of Gag proteins in and near the nucleus. The Gag proteins of retroviruses and retrotranspons have been detected in the nucleoplasm (MLV, PFV and RSV), in association with chromatin (PFV), in the nucleolus (FIV, HIV-1, MMTV, and RSV), at the nuclear rim (MPMV, Ty1, FIV) and at pericentrosomal sites (HIV-1 and PFV).

Historically, the Gag proteins of orthoretroviruses were thought to exist only in the cytoplasm of infected cells. However, it has recently been shown that Gag proteins of diverse retroviruses localize to the nucleus under specific conditions [19,20,24,30,72] (Figure 2). Demonstrating Gag nuclear trafficking has been difficult because only a small portion of the total cellular Gag population is detected in the nucleus under steady-state conditions. Despite this obstacle, the studies discussed herein have yielded important insights into how the intranuclear localization of Gag proteins could be involved in selection and encapsidation of the genomic RNA. 

The first retroviral Gag protein discovered to undergo active nucleocytoplasmic trafficking was RSV Gag, which has a well-understood mechanism of nuclear entry and egress. Whereas under steady-state conditions RSV Gag is detected primarily in the cytoplasm and along the plasma membrane, treatment of RSV-infected or Gag-expressing cells with the CRM1-inhibitor leptomycin B (LMB) dramatically concentrates Gag in the nucleus [20,170]. To gain insight into the role of RSV Gag in the nucleus, a genetic approach was undertaken. Viruses encoding Gag mutants that bypass the nucleus encapsidate reduced levels of genomic RNA and are noninfectious [156]. Reestablishment of Gag nuclear trafficking by inserting a heterologous nuclear localization signal (NLS) into the MA domain restores genome packaging to nearly wild-type levels [156]. In addition, *in vitro* evidence demonstrated that binding of Gag to nucleic acids facilitates the association with the CRM1-RanGTP export complex, suggesting that upon binding to nucleic acids in the nucleus, Gag is primed for export [25]. Ongoing experiments to visualize the Gag-viral RNA interaction suggest that Gag co-localizes with viral RNA in discrete subnuclear foci (Kaddis, Chiari-Fort and Parent, unpublished data). Together, these results support a model in which RSV Gag selects the genomic RNA for encapsidation in the nucleus (Figure 2). 

To dissect the mechanism by which RSV Gag undergoes transient nuclear trafficking, a series of experiments were performed to identify nuclear import and nuclear export signals (NES) in the RSV Gag polyprotein [20,26,170]. Two independent NLSs were found in the RSV Gag protein, one in the NC domain and the other in MA. The NLS in NC consists of a short stretch of basic residues, which is typical of a classical monopartite NLS. This NLS binds directly to the adapter protein importin α, which in turn recruits importin β to import the Gag monomer through the nuclear pore complex [24,25,26]. By contrast, the NLS in the RSV MA domain is atypical compared to other NLSs and resides within the N-terminal 86 residues of Gag [26]. Instead of containing a discrete cluster of basic residues like NC, there are 11 arginine and lysine residues dispersed throughout the sequence. However, in the tertiary structure of the N-terminal MA sequence, these amino acids form a basic patch, presumably to interact with the import factors transportin-3 (TNPO3) and importin-11 [26]. Interestingly, although TNPO3 imports serine-arginine rich (SR) splicing factors into the nucleus, MA does not contain a SR-rich region, indicating that the molecular basis underlying the TNPO3-Gag interaction is different from other TNPO3 cargoes. 

The MA domain does appear to be a critical determinant of RSV Gag function, as replacement of the RSV MA sequence with HIV-1 MA abrogates nuclear trafficking of the RSV/HIV chimera [35]. The RSV/HIV chimeric mutant virus was able to replicate at a reduced level in a single-round infectivity assay, suggesting that the HIV-1 MA domain may have a dominant effect, altering the trafficking of RSV Gag and the mechanism of RNA packaging. Furthermore, it should be noted that the RSV/HIV chimeric virus contained a reporter gene in place of the RSV *env* sequence, and the virus was pseudotyped with the vesicular stomatitis virus G envelope protein, which may have affected the infectivity results [35,171,172]. 

Why does RSV Gag contain two NLSs that interact with three different import factors? One possibility is that Gag encodes redundant signals to ensure it enters the nucleus. Alternatively, each NLS might be used selectively: the NLS that mediates entry of the Gag precursor during assembly could use one or more sets of import factors whereas the MA and NC NLSs in the mature proteins could interact with different importins during early infection. In support of this idea, insertion of a canonical NLS into MA (in addition to the atypical NLS already present) interferes with infectivity [173]. The replication defect does not involve budding, genome packaging, nuclear entry of the reverse transcription complex or proviral DNA synthesis; instead, the mutant virus does not undergo integration [173]. Thus, it is feasible that the nuclear import pathway followed by MA is crucial for successful integration, possibly by properly targeting the PIC. It is intriguing to speculate that the same principle could apply to RSV Gag during virus assembly: perhaps an importin bound to Gag directs it to a specific subnuclear location. For example, TNPO3 binds to the MA region of Gag and is also required to transport SR-protein splicing factors to splicing speckles [174,175,176]. If TNPO3 leads Gag to splicing speckles near sites of ongoing mRNA transcription [115], Gag would be close to the viral RNA as it is synthesized from the proviral DNA (Figure 1). We postulate that this strategy would be advantageous for Gag to preferentially select unspliced viral RNA for packaging as it is being transcribed, particularly since both spliced and unspliced RSV RNAs contain the *cis*-acting ψ-packaging sequence. Experiments to test this hypothesis are underway in our laboratory. 

## 6. Localization of Retroviral Gag Proteins to the Nucleus and Nuclear Envelope

**Figure 3:**
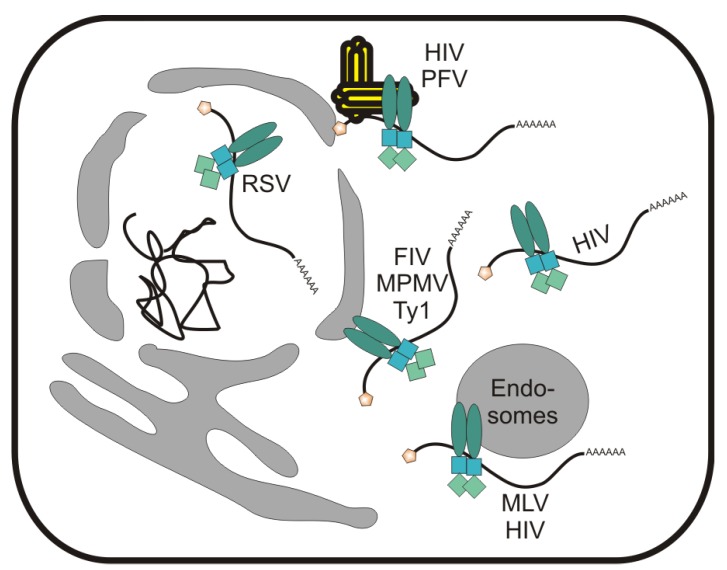
Subcellular sites of Gag-genomic RNA interaction where genomic RNA packaging may be initiated prior to plasma membrane localization. Retroviral Gag-RNA complexes have been visualized in pericentrosomal locations (HIV-1 and PFV) [168,188,189], on the cytoplasmic face of endosomes (HIV-1 and MLV) [190,191], on the cytoplasmic face of the nuclear envelope (FIV, MPMV, and Ty1) [19,33,72,192], and within the cytoplasm [193,194,195,196]. Genetic and biochemical data suggest that RSV Gag may bind its genomic RNA in the nucleus during transient nuclear trafficking of the Gag protein [20,26,156].

Although the mechanism of nuclear trafficking is the best understood for RSV Gag, other orthoretroviral Gag proteins also localize to the nucleus or at the nuclear rim (Figure 2 and Figure 3). The MLV Gag protein is primarily localized in the cytoplasm and at the plasma membrane, although a small nuclear pool (~18%) was detected using immunoelectron microscopy and biochemical fractionation [38]. The authors proposed that the nuclear fraction of MLV Gag might play a role in genome encapsidation or genomic RNA dimerization, and other studies support this hypothesis [177,178,179,180,181]. Experiments performed in MLV-infected cells treated with the transcription inhibitor actinomycin D demonstrated that unspliced viral RNA used for translation or packaging had different half-lives [182,183,184,185], suggesting that MLV may use different nuclear export pathways to distinguish translation-bound RNPs from encapsidated genomes. Interestingly, MLV genomic RNAs dimerize and are co-packaged preferentially when the genomic RNA is transcribed from nearby chromosomal sites [177,178,179,180,181], suggesting that MLV genomic RNA dimerizes co-transcriptionally within the nucleus. Elegant experiments elucidated the structural basis for preferential binding of the dimeric MLV RNA by showing that Gag binding sites on the RNA are exposed when dimerization occurs [186], thus dimerization and packaging are linked for MLV. Finally, murine Y RNAs selectively encapsidated into MLV particles are incorporated shortly after they are transcribed, raising the possibility that they may be recruited in the nucleus [187]. Considered together, these studies raise the possibility that selection of the dimeric genomic RNA and encapsidation of nuclear cellular RNAs are initiated by a small nuclear population of MLV Gag, as suggested by others [38,181,187]. Alternatively, it is feasible that one set of nuclear host factors binds to dimeric MLV genomes, transporting them to sites of virion assembly in the cytoplasm, whereas a different complement of cellular RNA transporters may direct monomeric unspliced RNA to ribosomes for translation. The merits of each model require further investigation. 

The lentiviral FIV Gag protein has a substantial degree of nuclear localization under steady-state conditions in feline cells, its natural host [19] (Figure 2). Nearly all of the FIV Gag protein became nuclear-localized when transfected or infected cells were treated with LMB, indicating that FIV Gag is a nucleocytoplasmic shuttling protein, much like RSV Gag. Although the NLS and NES sequences in FIV Gag have not been mapped, the LMB sensitive region of FIV Gag resides within the CA-NC-p2 sequence. When examined in HeLa cells, FIV genomic RNA and the Gag protein were observed at the cytoplasmic face of the nuclear envelope in a Rev- and ψ-dependent manner [72]. Deletion of the ψ sequence caused FIV RNA to be retained at the nuclear rim, whereas the Gag protein was localized to the plasma membrane. These findings suggest that the site of interaction between FIV Gag and the viral RNA may be the outer leaflet of the nuclear envelope (Figure 3). Kemler *et al*. raised the possibility that FIV Gag may encapsidate its RNA genome in the nucleus, but because the efficiency of export exceeds import, both Gag and the viral RNA genome appear to accumulate at the cytoplasmic leaflet of the nuclear membrane [72]. 

In the same study, HIV-1 genomic RNA also accumulated at the nuclear envelope [72] (Figure 3). However, the HIV-1 Gag protein was detected in the cytoplasm rather than at the nuclear rim, suggesting that the Gag-genomic RNA interaction may be initiated in the cytoplasm. Other studies reported that the pericentriolar microtubule organization center (MTOC) is a primary site of HIV-1 Gag-RNA interaction (Figure 3) [188,197]. The localization of HIV-1 RNA to the MTOC is mediated by the host protein hnRNPA2, which is subsequently involved in transporting the HIV-1 RNP to the plasma membrane for virion assembly [197,198]. The hnRNPA2 protein may initially bind to the unspliced HIV-1 RNA in the nucleus, where it plays a role in pre-mRNA processing and alternative splicing [199]. Thus, the MTOC may serve as a distribution center where HIV-1 Gag-RNA complexes interact with the motor protein dynein before travelling along the microtubule network to the plasma membrane [200]. In other studies of HIV-1 RNA trafficking in living cells, higher order Gag-RNA complexes were detected primarily at the plasma membrane, although smaller oligomeric complexes were found in the cytoplasm [194,196]. Therefore, although it remains unclear where HIV-1 Gag initially binds to the genomic RNA, there is general agreement that viral RNP formation occurs prior to plasma membrane localization (Figure 3).

Like FIV, a link may exist between MPMV Gag accumulation at the nuclear pore complex (NPC) and genomic RNA packaging (Figure 2 and Figure 3). The MPMV Gag protein interacts directly with the E2 SUMO conjugating enzyme Ubc9, which resides at the nuclear pore [34]. Additionally, overexpression of Ubc9 results in co-localization of Gag with Ubc9 at the nuclear rim, suggesting that nuclear trafficking of MPMV Gag may be a transient event mediated by interaction with Ubc9 [34]. MPMV Gag also accumulates in the nucleus with LMB treatment, indicating that it may undergo CRM1-dependent nucleocytoplasmic shuttling [35]. MPMV Gag mutants with alterations in the NLS in the pp24 domain no longer localize to the nuclear pore, and these mutant viruses are impaired in genomic RNA packaging [33]. Together, these observations suggest that MPMV Gag trafficking to the nuclear pore may be involved in viral RNA encapsidation. 

The yeast long terminal repeat (LTR) retrotransposons Ty1 and Ty3 have some trafficking properties in common with retroviruses. Ty1 and Ty3 encode Gag proteins that bind unspliced genomic RNA to form virus-like particles. Mex67p, the yeast ortholog of TAP/NXF1, is required for the export of Ty1 RNA from the nucleus [201]. In the cytoplasm, Ty1 RNA localizes with Gag to form cytoplasmic foci called T-bodies [201,202]. Recently it was shown that efficient export of Ty1 RNA from the nucleus is dependent on Gag [192]. Mutation of the *gag* initiation codon causes Ty1 RNA to accumulate in the nucleus, where it is degraded, suggesting that Gag stabilizes the RNA. Expression of Gag in *trans* restores Ty1 RNA nuclear export and RNA stability [192] (Figure 2 and Figure 3). These data suggest that Ty1 may use the Mex67p pathway for the bulk of Ty1 RNA nuclear export, whereas Gag may transport the subset of the RNA used as the genome during virus-like particle assembly in T-bodies. Ty1 Gag is not localized to the nucleus under steady-state conditions [201,202,203,204], but when expressed in a *mex67*-temperature sensitive strain, Gag accumulates at the nuclear envelope in a subset of cells [192]. Therefore, Gag nuclear localization appears to be transient, similarly to the nuclear trafficking properties of RSV Gag [20,23,24,25,156]. In addition, a mutant of Ty3 that is defective in RNA binding accumulates in the nucleus [205] and the Gag protein of Tf1, a retrotransposon of fission yeast, is nuclear-localized under steady-state conditions [206] (Figure 2). Thus, retroviruses and retrotransposons may share common mechanisms of Gag nuclear trafficking and unspliced RNA export, suggesting that comparative studies of retroelements are likely to yield important insights into novel mechanisms governing retrovirus particle assembly.

## 7. Localization of Gag to Subnuclear Sites

The Gag proteins of RSV and HIV-1 localize to the nucleolus under certain conditions [24] (Figure 2), and nucleolar trafficking of RSV Gag is dependent on basic residues in the NC domain. For RSV NC NoLS activity resides in residues 36–39 (KKRK), 61–63 (RKR) and 70R/73K. By contrast, the HIV-1 NC domain contains two independent NoLSs (R10/K11 and R32/K33/K34), each of which is sufficient for nucleolar localization of NC [24]. HIV-1 NC localizes to the nucleus during infection [57,58], but it is not clear whether nucleolar localization of the mature NC protein plays an important role during early infection. It is intriguing that many capsid and nucleic acid binding proteins from diverse viruses localize to the nucleolus, prompting the nucleolus to be referred to as “the gateway to viral infection" [19,24,26,39,57,58,207].

Due to the transient nature of its trafficking through the nucleus, detecting RSV Gag in nucleoli requires disruption of the nuclear export activity of Gag, either by mutating the p10 NES or treating infected cells with LMB [24]. The retention of Gag in nucleoli is decreased in the presence of the RSV Ψ packaging signal, suggesting that Gag binding to the packaging signal may either induce the Gag-RNA complex to leave the nucleolus or may prevent trafficking of the RSV RNP through the nucleolus. Whether there is a host protein or RNA that binds to RSV Gag-RNA complexes in the nucleolus is unknown. A subset of the HIV-1 Gag protein also localizes to nucleoli when the provirus is expressed at high levels from an inducible, Rev-dependent, integrated provirus. HIV-1 Gag accumulates in nucleoli when co-expressed with Rev or NC, and positive FRET (fluorescence resonance energy transfer) was observed between Gag and Rev, indicating an intimate association between these proteins within the nucleolus [24]. Whether the nucleolar localization of HIV-1 Gag or the interaction between Gag and Rev has any significant role in genomic RNA encapsidation or virus replication remains to be determined. 

Other retroviral Gag proteins have been reported to localize to the nucleolus under certain conditions. For example, LMB treatment of transfected cells causes FIV Gag to accumulate in nucleoli when expressed alone and nucleolar localization was dependent on NC [19] (Figure 2). However, the importance of Gag or NC nucleolar localization was not further investigated. In addition, MMTV Gag is partially nucleolar-localized in a murine mammary cell line chronically infected with a highly tumorigenic strain of the virus [30]. Furthermore, a subset of MMTV Gag can be induced to accumulate in the nucleolus with overexpression of ribosomal protein L9, which interacts with MMTV Gag in an extraribosomal context (Figure 2). A functional role for this interaction was demonstrated by knockdown of L9, which causes a decrease in MMTV particle production [30]. Thus, it is possible that nucleolar trafficking of MMTV Gag is an important step in virus assembly pathway, although further experiments will be needed to test this intriguing idea.

In addition to localizing to the nucleolus, the RSV Gag protein also accumulates in discrete nucleoplasmic foci when restricted to the nucleus by mutating the NES or inhibiting CRM1-mediated nuclear export [20,23] (Figure 2). It will be interesting to determine whether RSV Gag is associated with a particular subnuclear body or tethered to a specific cellular protein or RNA at these sites. The appearance of these subnuclear foci of RSV Gag is similar to the localization of *Spumaretrovirus* PFV Gag in nucleoplasmic foci during prophase [82]. In the case of PFV, it was recently discovered that the Gag protein binds to the host chromosome in mitotic cells using a chromatin-tethering domain encoded in the glycine-arginine box II domain, which binds to core histones H2A and H2B [82]. The PFV Gag-chromatin interaction facilitates proviral integration, although the precise mechanism has not been defined. In addition, a CRM1-dependent nuclear export pathway was reported for PFV Gag, although this finding is controversial [21,83]. An intriguing idea not yet experimentally tested is whether the association PFV Gag with the proviral integration site [82] could position Gag at the site of PFV RNA synthesis to facilitate selection of genomic RNA. Additional studies are required to determine whether PFV Gag nuclear localization plays a role in genomic RNA encapsidation or whether its function is restricted to early events, when it facilitates integration of the provirus.

## 8. Remaining Questions about Nuclear Trafficking during Retrovirus Assembly

In the past decade, great strides have been made in understanding the mechanisms that guide nuclear import, nuclear export, and the intranuclear activities of retroviral and retrotransposon Gag proteins. The use of a comparative approach to identify common and unique features of different Gag proteins in the nucleus has been very illuminating. Although it is not yet clear whether all Gag proteins transit through the nucleus at some low level, examination of the roles of nucleocytoplasmic trafficking in those Gag proteins that do enter the nucleus or associate with the nuclear envelope (RSV, MPMV, MLV, HIV-1, FIV, PFV, Ty1 and Tf1) are important to pursue. 

Is Gag nuclear trafficking involved in genomic RNA packaging? To date, studies of RSV, MPMV, and Ty1 support this idea. PFV Gag associates with chromatin and plays a role in integration, although it might also function in genome encapsidation of nuclear transcripts [21,83]. In simple retroviruses, there could be a temporal switch with unspliced viral RNA export mediated by Tap/NXF1 for translation of structural proteins early after integration. As Gag proteins accumulate, they could enter the nucleus and export viral RNA for packaging. Thus, could there be two pathways of nuclear export of unspliced viral RNAs, or two distinct viral RNPs, one primed for packaging and the other for translation? If so, it is logical to suggest that the cytoplasmic fates of retroviral RNAs may be pre-determined in the nucleus according to the composition of the RNP.

Finding that several retroviral Gag proteins undergo nucleolar localization raises intriguing questions about whether there is a unifying function of the nucleolus in virus replication. One possibility is that retroviral Gag proteins interact with a host protein or RNA in the nucleolus involved in genome packaging or virus assembly. It is intriguing that several RNA pol III transcripts are enriched in retroviral particles, including RNAs that are processed in the nucleolus such as tRNA, U6 small nuclear RNA, 7SL [208,209], and 5S rRNA, [187,210,211,212,213,214,215,216]. Whether these RNAs are recruited into virus particles as passengers encountered along the subcellular trafficking route of Gag or whether they play a facilitating role in virus replication remains to be seen. As an interesting connection with endogenous retroelements and the nucleolus, there is strong genetic evidence that the nucleolus may be the site of RNP maturation for the LINE (long-interspersed nuclear element) retrotransposons, with U6 potentially playing a central role [217]. Thus, further investigation into the role of nuclear machinery in retrovirus and retrotransposon RNA processing, nuclear export, and RNP trafficking may generate new paradigms about genomic RNA encapsidation and virus particle assembly.
